# Vibration Position Detection of Robot Arm Based on Feature Extraction of 3D Lidar

**DOI:** 10.3390/s24206584

**Published:** 2024-10-12

**Authors:** Jinchao Hu, Xiaobin Xu, Chenfei Cao, Zhenghong Tian, Yuanshan Ma, Xiao Sun, Jian Yang

**Affiliations:** 1 College of Mechanical and Electrical Engineering, Hohai University, Changzhou 213200, China; uuleslie@gmail.com (J.H.); 211319010001@hhu.edu.cn (C.C.); 2 College of Water Conservancy and Hydropower Engineering, Hohai University, Nanjing 210098, China; zh-tian@hhu.edu.cn (Z.T.); 180402020006@hhu.edu.cn (Y.M.); xiaos@hhu.edu.cn (X.S.); 3 College of Mechanical Engineering, Yangzhou University, Yangzhou 225127, China; jianyang@yzu.edu.cn

**Keywords:** concrete vibration, 3D point cloud, projection, computer vision

## Abstract

In the process of construction, pouring and vibrating concrete on existing reinforced structures is a necessary process. This paper presents an automatic vibration position detecting method based on the feature extraction of 3D lidar point clouds. Compared with the image-based method, this method has better anti-interference performance to light with reduced computational consumption. First, lidar scans are used to capture multiple frames of local steel bar point clouds. Then, the clouds are stitched by Normal Distribution Transform (NDT) for preliminary matching and Iterative Closest Point (ICP) for fine-matching. The Graph-Based Optimization (g2o) method further refines the precision of the 3D registration. Afterwards, the 3D point clouds are projected into a 2D image. Finally, the locations of concrete vibration points and concrete casting points are discerned through point cloud and image processing technologies. Experiments demonstrate that the proposed automatic method outperforms ICP and NDT algorithms, reducing the mean square error (MSE) by 11.5% and 11.37%, respectively. The maximum discrepancies in identifying concrete vibration points and concrete casting points are 0.059 ± 0.031 m and 0.089 ± 0.0493 m, respectively, fulfilling the requirement for concrete vibration detection.

## 1. Introduction

Reinforced concrete is commonly employed in the field of engineering construction. Vibrating poured concrete is essential to achieve uniformity and proper density in the concrete, thereby improving the strength and other performance indicators of the reinforced concrete [1,2,3]. The conventional approach involves manual vibration, which requires pre-planning of vibrating spacings [4,5]. However, this method relies heavily on the operator’s expertise, leading to frequent adjustments in vibrating positions based on experience. As it involves continuous physical effort, the energy generated by vibration fluctuates due to the manual configuration of various vibration positions and spacings [6]. Such inconsistency in concrete vibration quality can result in vibration leakage, under-vibration, and over-vibration, adversely affecting the project’s quality.

With advancements in information technology, sensor technology, and data processing, automation and computer vision are increasingly adopted in engineering construction. This signifies a shift from the conventional manual construction methods towards intelligent construction. Notably, these technologies have already found applications in concrete quality testing [7,8], concrete pouring [9,10,11,12], and concrete vibration [13,14,15].

The vibration of concrete plays a critical role in the construction process, and the identification of concrete vibration locations is a crucial aspect of intelligent concrete vibration. In the process of construction, pouring and vibrating concrete on the existing reinforced structure is a necessary process. The environment for vibrating concrete is typically under the layout and configuration of reinforcement bars, which vary in diameter and spacing. Therefore, enhancing the functionality of identifying concrete vibration points under this environment holds considerable significance in promoting intelligent concrete vibration.

Tian designed a system for visualizing concrete vibration [16]. This system investigates the radius of action by varying the slump of the concrete mix, the aggregate gradation, and the vibration duration. Meanwhile, the system transmits positioning and vibration data to the server, enabling real-time 3D visualization of concrete vibration. Li developed a vibration energy transfer model based on the vibrator’s operational mechanism and the theory of vibration energy [6]. Using the 3D model, they generated a vibration energy distribution map to aid in the on-site selection of concrete vibration points. Although this approach reduces the manual effort needed for identifying vibration points, it still requires manual assistance. To further minimize manual intervention, researchers are investigating the application of image processing and neural networks for the identification of concrete vibration points.

Numerous vision-based methods for intelligent concrete vibration have been proposed. Wang introduced a global location system (GNSS) positioning information denoising method based on Kalman filtering [17]. Improved Faster R-CNN and DeblurGAN-v2 networks were employed during the concrete pouring process, promoting the development of intelligent concrete vibration through the 3D perception of construction information. Chen developed an autonomous vibration robot system based on machine vision. The system intelligently identifies concrete surface images, providing state information about the concrete that can be used in automatic vibration operations [18]. Cui identified the surface of concrete to be vibrated by using an enhanced CNN neural network and real-time vibration monitoring technology based on the Internet of Things (IoT) [19]. Ren used semi-supervised learning and data enhancement techniques in concrete vibration detection and improved recognition accuracy as well as detection efficiency [20]. Quan integrated the HNN recognition model of CNN and RNN to achieve the real-time monitoring of concrete vibration through the analysis of collected image and signal data [14]. However, these vision-based methods have limitations, including low accuracies in complex scenes and susceptibility to light interference.

In the process of vibrating concrete pouring for reinforced concrete, although image-based methods (such as classical image processing and deep learning) can provide certain detection capabilities, their accuracy and reliability are often affected by variations in ambient lighting. The adoption of LiDAR detection methods allows for the capture of high-density three-dimensional point cloud data, which can accurately depict the distribution and position of rebars. This effectively compensates for the shortcomings of image-based methods in conditions of insufficient lighting or interference.

Lidar is used to construct a high-precision 3D model of the target, making it well-suited for tasks in construction engineering [21,22] and detection [23,24], facilitating high-precision detection of reinforced concrete. To address the challenge of detecting rebar spacing onsite before concrete pouring, Yuan applied mobile lidar technology to create a 3D representation of rebar layouts in reinforced concrete structures [25]. By calculating the relative bending moment index, they attained precise local rebar detection, offering an objective assessment of rebar layout quality. Subsequently, Yuan applied projection and slicing algorithms to extract the positions of bridge concrete and rebar, achieving automatic local inspection with high precision [26]. This method effectively reduces the time and manual workload of detecting rebar spacing before concrete pouring. However, these methods can only identify local information about rebar spacing during concrete pouring and vibration and still require manual verification and detection. They lack the capability to autonomously identify concrete vibration points and concrete casting points.

In a complex construction site, local point cloud data can be gathered only within a limited range. Point cloud data collected from a far range lacks details, leading to misjudgment and overlooking of the vibrating area. Moreover, direct processing of the point cloud data requires substantial computing resources. To address these issues, this paper proposes an autonomous vibration method implemented on a vibrating robot arm using 3D features extracted by lidar. The contributions of this article are as follows:

(1) A lidar calibration method based on feature point constraints for a vibration robot is proposed.
(2) A high-precision rebar array reconstruction method is introduced based on the fusion of Normal Distribution Transform-Iterative Closest Point (NDT-ICP) and graph optimization.
(3) A technique for positioning concrete vibration points and concrete casting points is presented, based on pseudo-visuals from 3D point cloud projection.

The proposed method considers the drawbacks of image-based methods and through dimensionality reduction and image processing methods. We avoid the problem of a large number of operations caused by the direct use of point cloud data detection. The detection accuracy and operation speed are guaranteed. The remainder of this article is structured as follows: Section 2 introduces the algorithm process and key technical points. Section 3 conducts splicing ablation experiments and compares the calculated results with the actual insertion points. Section 4 provides the conclusion.

## 2. Vibration Position Detection Based on Feature Extraction of 3D Lidar

**Figure 1:**
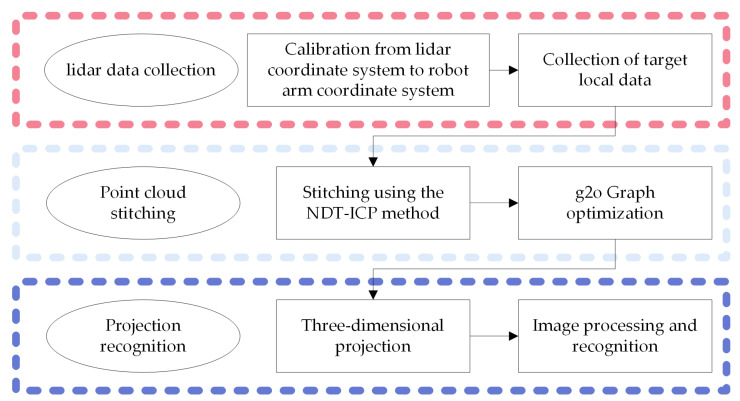
Algorithm framework diagram.

The proposed lidar-based autonomous vibration method is implemented on a vibrating robot arm. The overall algorithm block diagram is depicted in Figure 1. First, the lidar scans the target object at multiple angles to obtain multi-segment point clouds. The collected 3D point clouds are spliced together to generate a comprehensive 3D point cloud of the overall target object. Then, this 3D point cloud is projected onto a plane, converting the result into a 2D image. Subsequently, by employing image processing technology, the concrete vibration area and concrete distribution area are identified, and their central point positions are determined. Finally, the recognition results are back-projected into the 3D coordinate system.

### 2.1. Hand-Eye Calibration

The vibrating robot arm employs lidar as its sensing device. It is crucial to accomplish the calibration of the lidar frame to the robotic arm frame to achieve precise vibration [27]. This article adopts a nonlinear optimization method for calibration, with the process outlined as follows:

Assume the lidar point set as $L=\{l_{1},l_{2},\cdots \cdots ,l_{n}\}$, $l_{1}=(x_{l_{1}},y_{l_{1}},z_{l_{1}})$ and the robot arm point set as $M=\{m_{1},m_{2},\cdots \cdots ,m_{n}\}$, $m_{1}=(x_{m_{1}},y_{m_{1}},z_{m_{1}})$, then

(1) M=TL

where T the transformation matrix of the two coordinate systems. Assume the error equation is

(2) f(T)=d

where f represents the error function, which is constructed using point sets ***L*** and ***M*** as input and output, respectively, and *d* denotes the distance for each pair of points. Compute the Jacobian matrix of f with respect to ***T***, denoted as $J=\frac{\theta f}{\theta T^{2}}$, and employ a nonlinear iterative solution method to minimize *d*, approaching it towards zero.

(3) $$T\leftarrow T-{\left(J^{T}J+\lambda diag(J^{T}J)\right)}^{-1}J^{T}d$$

where λ is the factor determined by ***M***.

**Figure 2:**
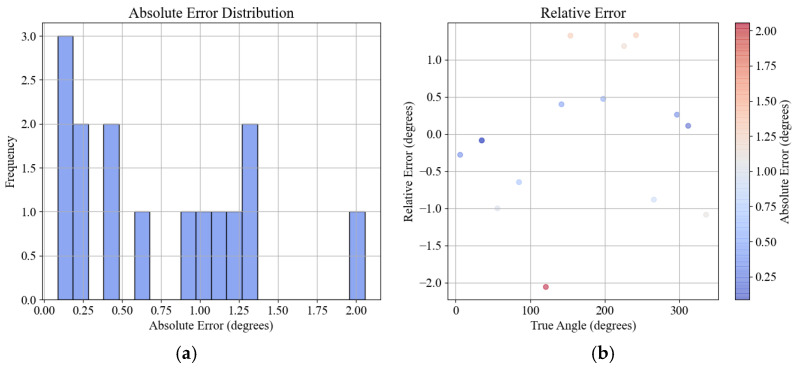

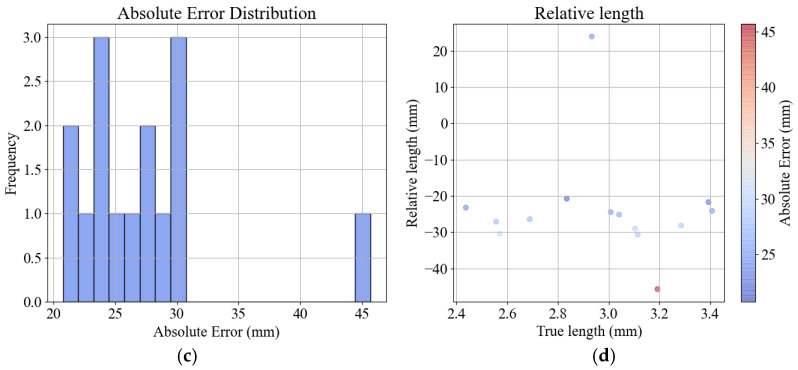
Calibration error plots: (**a**) calibration error histogram; (**b**) calibration error heat map; (**c**) length error histogram; (**d**) length error heat map.

The error diagram of the true value and the measured point in the robot’s coordinate system is depicted in Figure 2. As can be seen from the histogram frequency diagram 2(a) and the heat diagram 2(b), the calibration angle errors are predominantly within the range of 0 degrees and 1.25 degrees, with the error distribution mostly between 0 degrees and 0.25 degrees. From the histogram frequency diagram 2(c) and the heat diagram 2(d), it is evident that the calibration distance errors are mostly between 20 mm and 30 mm. Therefore, the calibration results obtained via nonlinear optimization exhibit smaller errors, making them suitable for practical applications.

### 2.2. Point Cloud Matching Method Based on NDT-ICP and Graph Optimization

Lidar is used for the local data collection of steel bars. To obtain global data of steel bars, the NDT-ICP method is employed to stitch the point clouds of steel bars from multiple viewpoints. This process comprises two steps: NDT [28] for coarse registration and ICP [29] for fine registration. To enhance stitching accuracy, the robotic arm’s rotation angle is used as the initial value to improve the precision of point cloud matching. The pose transformation matrix obtained in the NDT serves as the initial pose value in the ICP. The ICP fine registration process employs the Gauss-Newton method to iteratively solve the objective function, outputting the optimal transformation matrix. Finally, the g2o [30] algorithm is utilized to optimize the stitching results and minimize the errors caused by stitching through global optimization.

**Figure 3:**
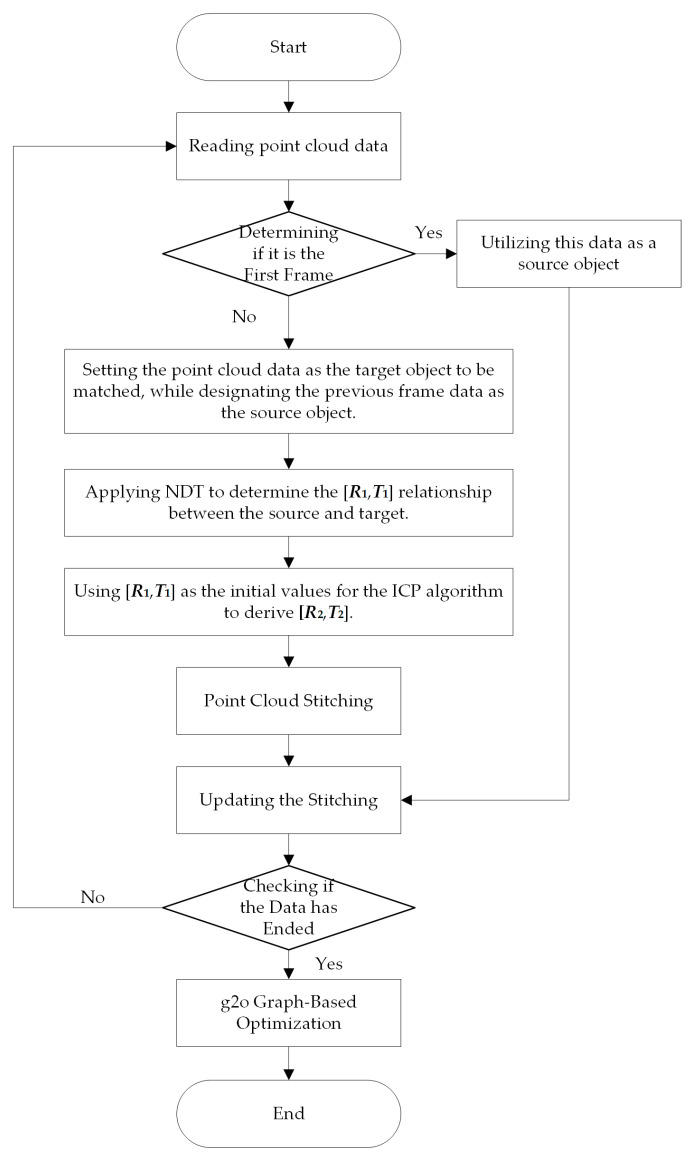
Flow chart of matching method.

The algorithm flow is illustrated in Figure 3. The first step involves reading the point cloud data and determining whether it is the first frame. If so, the data is used directly as the source object for the next frame; otherwise, it is set as the target object. Then, NDT is used to calculate the pose transformation matrix [*R*_1_, *T*_1_] of the source and target (where *R* represents the rotation pose transformation, and ***T*** is the translation pose transformation). The result is used as the initial value of the ICP matching, and the solution is [*R*_2_, *T*_2_]. The solution obtained is then used to stitch the source point cloud and the target point cloud, subsequently updating the global point cloud. Once all the data has been stitched, g2o global Graph-Based Optimization is conducted.

#### 2.2.1. NDT Algorithm

The NDT registration algorithm segments the reference point cloud into a grid, computes the probability density function based on the points within each grid cell, and determines the mean and covariance matrix of the multidimensional normal distribution. When registering a new point cloud, it is transformed to align with the grid of the reference point cloud. The calculated probability density function for each point is calculated as follows:

(4) $$v(x)=\frac{1}{b}\exp[-\frac{{\left(x-\mu \right)}^{T}C^{-1}(x-\mu )}{2}]$$

where ***µ*** is the mean vector within the voxel grid containing the point cloud ***x***, ***C*** represents the covariance matrix within the same voxel grid, and ***b*** is a constant ***µ***. ***C*** within each voxel grid can be defined as

(5) $$\mu =\frac{1}{h}\sum_{i=1}^{h}x_{i}$$

(6) $$C=\frac{1}{h-1}\sum_{i=1}^{h}(x_{i}-\mu ){\left(x_{i}-\mu \right)}^{T}$$

where ***x****_i_* (*i* = 1, 2, …, *h*) are all point clouds in the grid.

The NDT registration objective function is derived by summing the probability density calculated for each grid. In the process of summing the probability distribution of each mapping point, the formula for calculating *s*(***p***) is

(7) $$s(p)=\sum_{i=1}^{h}v[T(p,x_{i})]=\sum_{h}\exp\left[-\frac{{\left(x_{i}^{\prime }-\mu_{i}^{\prime }\right)}^{T}{C^{\prime }}^{-1}(x_{i}^{\prime }-\mu_{i}^{\prime })}{2}\right]$$

In the formula, the initial transformation parameter ***p*** = [***t***|***r*|*φ***], where ***t*** = [*t*_x_, *t*_y_, *t*_z_], ***r*** = [*r*_x_, *r*_y_, *r*_z_], $x_{i}^{\prime }$ is the position of ***x*** in the target point cloud coordinate system obtained through the transformation matrix, $u_{i}^{\prime }$ represents the corresponding mean vector of $x_{i}^{\prime }$, and $C^{\prime }$ represents $x_{i}^{\prime }$, the covariance matrix of the corresponding grid. The 3D transformation matrix ***T***(***p***, ***x***) can be expressed as

(8) $$T(p,x)=\left[\begin{array}{ccc} or_{x}^{2}+c & or_{x}r_{y}-sr_{z} & or_{x}r_{y}+sr_{y}\\ or_{x}r_{y}+sr_{z} & or_{y}^{2}+c & or_{y}r_{z}+sr_{x}\\ or_{x}r_{y}-sr_{y} & or_{y}r_{z}-sr_{x} & or_{z}^{2}+c \end{array}\right]$$

where *s* = sin*φ*, *c* = cos*φ*, and *o* = 1 − *c*.

Newton’s optimization algorithm is used to optimize the objective function—that is, to find the transformation parameter ***p*** that minimizes the objective function. Let *f* = *s*(***p***), and to minimize the function *f*, the following equation processing must be performed for each operation:

(9) HΔp=−g

where **Δ*p*** is the change of the transformation parameter, ***g*** is the transpose gradient of *f*, and its elements can be expressed as

(10) $$g_{i}=\frac{\partial f}{\partial p_{i}}$$

where ***p****_i_* and ***p****_j_* are transformation parameters, and ***H*** is the Hessian matrix of *f*, whose elements can be expressed as

(11) $$H_{ij}=\frac{\partial^{2}f}{\partial p_{i}\partial p_{j}}$$

#### 2.2.2. g2o Graph-Based Optimization

Pose optimization involves solving the nonlinear least squares problem. The g2o framework solves it based on graph optimization. It is a back-end optimization performed when the pose graph is known.

The pose convention of the subgraph is $p=(p_{1},p_{2},\cdots ,p_{n})$. In the graph optimization stage, it is necessary to optimize the poses of these nodes. Each node pose can be expressed as follows:

(12) $$p_{i}={\left({\dot{x}}_{i},{\dot{y}}_{i},{\dot{z}}_{i}\right)}^{T}$$

where $T_{i}=\left[\begin{array}{cc} R_{i} & t_{i}^{T}\\ 0^{T} & 1 \end{array}\right]$, $t_{i}={\left({\dot{x}}_{i},{\dot{y}}_{i},{\dot{z}}_{i}\right)}^{T}$ represents the position coordinates (translation vector) in the map, and $R_{i}$ represents the direction angle in the map.

In the loop detection process, the relative pose between the node ***p****_i_* and the node ***p****_j_* is obtained by matching the estimation as the observation value ***m****_ij_*, which is expressed as follows:

(13) $$m_{ij}=T_{ij}$$

The nodes ***p****_i_* and ***p****_j_* can be measured by the odometer. The predicted value ***m****_ij_’* can be defined as

(14) $$m_{ij}^{\prime }(p_{i},p_{j})=P_{i}^{-1}P_{j}$$

where ***P****_i_* represents the transformation matrix corresponding to node ***p****_i_*, and ***P****_j_* represents the transformation matrix corresponding to node ***p****_j_*_._

The error function is defined as

(15) $$e_{ij}(p_{i},p_{j})=T2v(M_{ij}^{-1}M_{ij}^{\prime })$$

where ***M****_ij_* and $M_{ij}^{\prime }$ are the transformation matrices corresponding to ***m****_ij_* and $m_{ij}^{\prime }$, respectively, and *T*2*v* represents the transformation matrix corresponding to the corresponding pose.

### 2.3. Projection of Steel Bars and Identification of Vibration Concrete Casting Points

**Figure 4:**
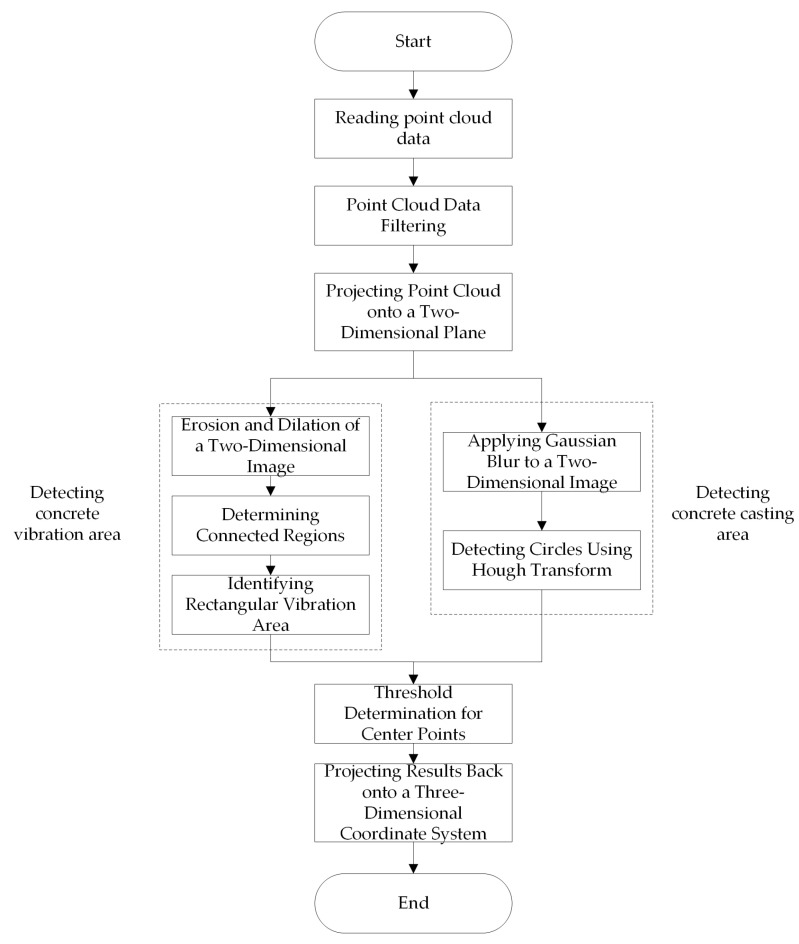
Point cloud processing flow chart.

Given the substantial data volume of stitched steel bar point clouds, direct usage for identifying concrete vibration points and concrete casting points would be time-consuming. To enhance detection efficiency and accuracy, the 3D point cloud processing algorithm is proposed and depicted in Figure 4. First, the stitched point cloud data is read and preprocessed using pass-through filtering, radius filtering, and statistical filtering. Then, the 3D cloud is projected onto a plane and converted into a 2D image. Subsequently, rectangular vibrating areas and circular concrete casting areas are identified on the 2D image. To identify a circular concrete casting area, a Gaussian blur is used to smooth the distinctive edge features of the projected image. Hough transform detection is then employed to obtain circular area information. To identify a rectangular vibration area, the projected 2D image is first dilated and eroded to eliminate image noise and fill in small spaces within the object. By applying the connectivity analysis, each connected area is associated with a bounding rectangle, and adjacent bounding rectangles intersect. The filling value of the bounding rectangle is set to 255. After the identification of these areas, by setting a distance threshold between the center points of the casting area and vibrating area, duplicate center points are removed. Finally, the identified concrete vibration points and concrete casting points are back-projected to obtain the 3D spatial coordinates.

The projection principle is as follows: since a point cloud is composed of a set of unordered points in 3D space, it can be projected onto any 2D plane.

Assuming a plane in 3D space is

(16) Ax+By+Cz+D=0

If $\left(x_{1},y_{1},z_{1}\right)$ and $\left(x_{2},y_{2},z_{2}\right)$ are the two points on the plane, then

(17) $$A\left(x_{2}-x_{1}\right)+B\left(y_{2}-y_{1}\right)+C\left(z_{2}-z_{1}\right)=0$$

where $\left(x_{2}-x_{1},y_{2}-y_{1},z_{2}-z_{1}\right)$ is the vector on the plane and is perpendicular to (A,B,C), thereby (A,B,C) is the normal vector of the plane.

For any point *p* = $\left(x_{0},y_{0},z_{0}\right)$, it is projected onto the plane with the coordinates $p^{\prime }=(x^{\prime },y^{\prime },z^{\prime })$, following the rule that *pp*’ is parallel to (A,B,C):

(18) $$\frac{x-x_{0}}{A}=\frac{y-y_{0}}{B}=\frac{z-z_{0}}{C}=t$$

which can be reorganized as

(19) $$\left\{\begin{array}{c} x=At+x_{0}\\ y=Bt+y_{0}\\ z=Ct+z_{0} \end{array}\right.$$

By substituting Equation (19) into Equation (16), *t* can be written as

(20) $$t=-\frac{Ax_{0}+By_{0}+Cz_{0}+D}{A^{2}+B^{2}+C^{2}}$$

By substituting Equation (20) into Equation (19), the projected coordinates can be obtained:

(21) $$\left\{\begin{array}{c} x_{0}=x+A\frac{Ax_{0}+By_{0}+Cz_{0}+D}{A^{2}+B^{2}+C^{2}}\\ y_{0}=y+B\frac{Ax_{0}+By_{0}+Cz_{0}+D}{A^{2}+B^{2}+C^{2}}\\ z_{0}=z+C\frac{Ax_{0}+By_{0}+Cz_{0}+D}{A^{2}+B^{2}+C^{2}} \end{array}\right.$$

## 3. Experiments and Analysis

**Figure 5:**
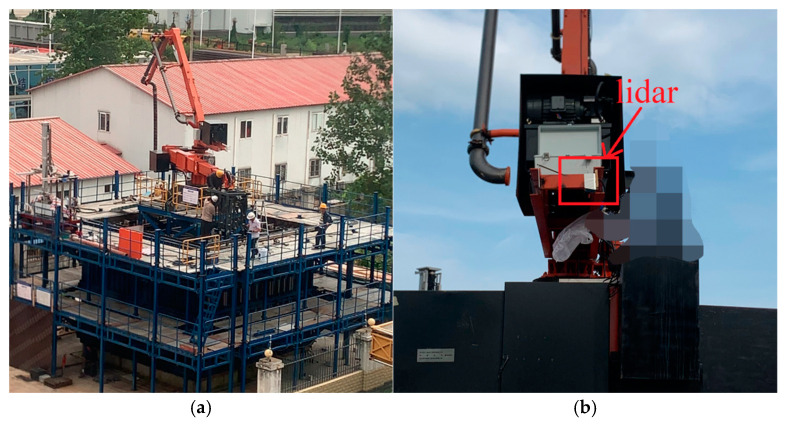

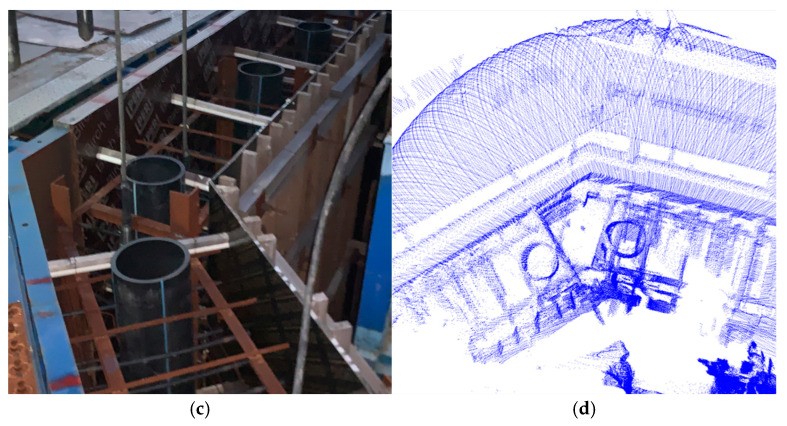
Actual scenario: (**a**) actual construction scenario; (**b**) lidar installation position; (**c**) local scenario; (**d**) local point cloud.

The experimental setup is shown in Figure 5a. Livox Mid-70 lidar is employed in the setup, featuring a circular Field of View (FOV) of 70.4 degrees, a near blind zone of 5 cm, and a maximum detection range of 260 m. Its ranging accuracy is 3 cm, and the accuracy within 20 m is 2 cm. The lidar is mounted at the end of a vibrating arm, as illustrated in Figure 5b. The experimental platform consists of two stories. The lidar is oriented vertically downward to capture steel bar data in a clockwise direction. The local steel bar map is presented in Figure 5c, and its single-frame point cloud data is shown in Figure 5d. The point cloud is consistent with the real object, including the concrete pouring bucket and steel bar.

### 3.1. Point Cloud Projection Splicing Experiment

The lidar collects 51 frames of point cloud data as it moves with the robotic arm in one circular motion. The splicing algorithm in this paper utilizes a graph optimization method based on NDT-ICP, with a comparison to the conventional algorithms of NDT and ICP. Mean Square Error (MSE), from the Point Cloud Library (PCL), is used to evaluate the quality of point cloud matching. MSE is a metric indicating the degree of discrepancy between the estimated and the actual values. The results are presented in Table 1. It can be seen that compared to ICP, NDT, and NDT-ICP, the proposed algorithm reduces the error by 11.5% (0.0016107), 11.37% (0.0015907), and 11.04% (0.0015388), respectively. Through a splicing method combining NDT’s coarse match and ICP’s precise match, the errors are reduced by 0.33% and 0.46%, respectively, compared to the NDT and ICP algorithms. Therefore, utilizing the NDT-ICP matching method can effectively reduce the splicing error, while using the g2o map (Paper Algorithm) optimization strategy can further minimize these errors.

**Figure 6:**
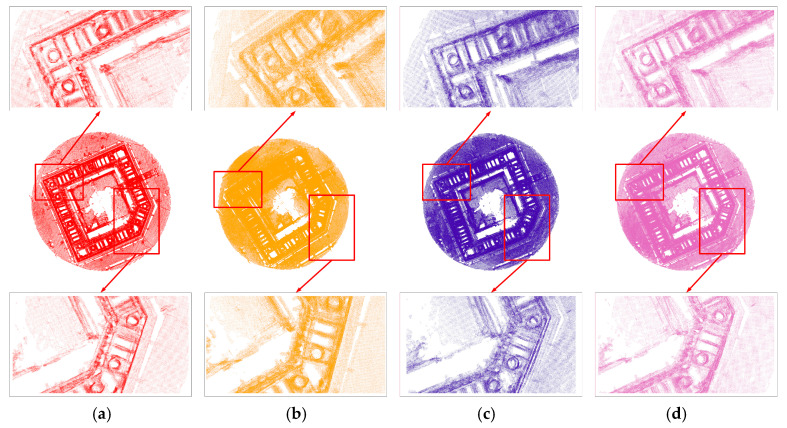
Algorithm point cloud comparison chart: (**a**) proposed algorithm; (**b**) NDT-ICP; (**c**) ICP; (**d**) NDT.

The point clouds processed by different algorithms are displayed in Figure 6. It can be seen that the proposed algorithm, ICP, and NDT can effectively reconstruct the 3D model, while NDT-ICP is imperfect. For NDT and ICP, ghosting presents at the corner of the rebar positions, while the proposed algorithm produces a clearer result. At the right angle, the steel bars spliced by the ICP and NDT algorithms are slightly distorted, whereas the proposed algorithm avoids such distortion. This can be explained by the sensitivity of ICP to the initial match value, as it relies on point-to-point distance for matching. On the other hand, NDT employs rasterization to reduce the impact of noise during splicing, making it more robust than ICP when rotation is involved. In the closed-loop area, the proposed algorithm combines the strengths of NDT and ICP and additionally integrates closed-loop detection and overall graph optimization. This reduces the cumulative error associated with matching multiple frames using NDT-ICP. Consequently, in local areas, compared to other algorithms exhibiting ghostlings, the proposed algorithm produces a clearer result and a higher reconstruction accuracy.

### 3.2. Point Cloud Projection Recognition Experiment

**Figure 7:**
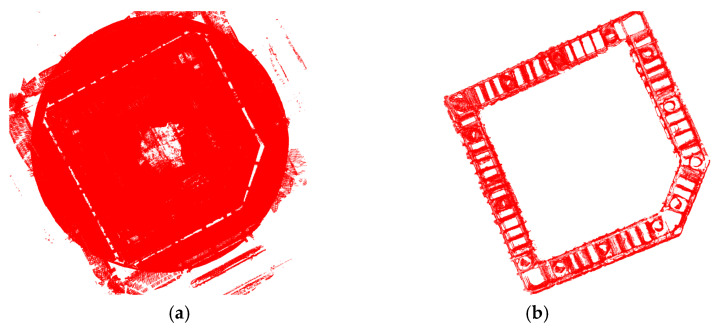
Comparison between the original and filtered point cloud: (**a**) original point cloud; (**b**) filtered point cloud.

The spliced point cloud comprises densely scattered points, as shown in Figure 7a. Direct detection performed on such a dense point cloud results in a prolonged detection time and a considerable reduction in real-time performance. Straight-through filtering is employed to coarsely filter out the invalid area, while radius filtering and statistical filtering are used to further remove discrete points. The radius of the radius filter (RadiusSearch) is set to 0.05, and the minimum number of neighboring points (MinNeighborsInRadius) is set to 20. The average number of points (MeanK) for the statistical filter is set to 15, and the standard deviation multiplier threshold (StddevMulThresh) is set to 1.0. The filtered point cloud is presented in Figure 7b. It is clear that the data volume of the original point cloud is extensive, presenting challenges in identifying the rectangular concrete vibration area and the circular concrete casting area. The direct projection of the original data onto the 2D plane results in excessive point density. In contrast, the filtered point cloud displays distinct rectangular and circular features with fewer cluttered points, making it suitable for subsequent projection and image processing.

**Figure 8:**
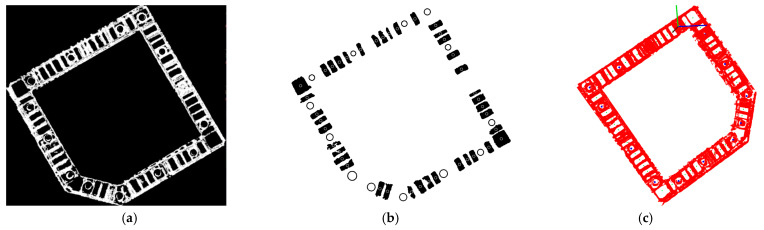
Image processing: (**a**) Gaussian blur result; (**b**) recognition of rectangles and circles; (**c**) results in point cloud. (The red axis is the X-axis, the green axis is the Y-axis, and the blue axis is the z-axis).

The filtered point cloud is projected onto the plane-by-plane fitting, transforming it into a 2D image. However, the distinctive edge features of the projected 2D image could lead to misrecognitions. Therefore, a Gaussian blur is applied to smooth the image edges, followed by Hough transform to identify the circular holes. The kernel size and sigma for the Gaussian blur are set as 5 and 1.0. The parameters for the Hough transform detection circle are configured with a radius range of 8 m to 20 m and param = 18. As illustrated in Figure 8a, the point cloud data processed by the Gaussian blur exhibits smoother circular holes and rectangular edges, effectively avoiding misidentifications.

For the projected 2D image, corrosion, expansion, and connected domain judgment methods are utilized to extract the minimum enclosing rectangular frame to detect the rectangular vibration area. The connected domain area is evaluated and invalid results are eliminated. Figure 8b displays the concrete vibration and concrete casting results after identification. The results demonstrate successful identification of all concrete casting points and concrete vibration points, except for the initially occluded ones.

**Figure 9:**
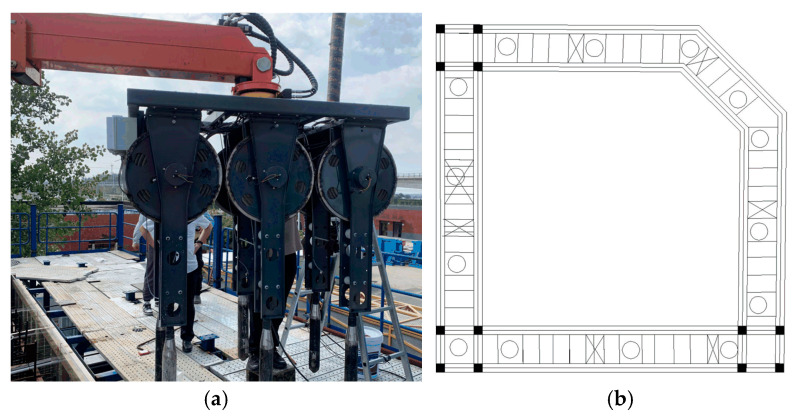
Vibrator and CAD drawing: (**a**) vibrators; (**b**) CAD drawing.

The identified concrete vibration points and concrete casting points are projected back into the 3D coordinate system, where the blue points represent casting points and the green points denote vibration points. These results are shown in Figure 8c. Figure 9a presents the vibrator rod group in actual construction. The distance between vibration points depends on the diameter of the vibrator rod. In this study, the diameter of the vibrator rod is 80 mm, and the radius of action ranges from seven to eight times of that diameter. Comparing the vibration and concrete casting results in Figure 8c, where it is evident that the vibration area and concrete casting area calculated by the proposed algorithm meet the vibration requirements. The accuracy of the vibration and casting points is determined by comparing the CAD drawing of the actual construction and the insertion point calculated by the proposed algorithm. Figure 9b displays the CAD drawing, where the area marked with cross represents blocked areas. Given the presence of steel bars and obstructions in such areas, the insertion of vibration and casting points is not viable. The plane scale is 1:1. The actual number of insertion points in the vibration area is 40, and the actual number of insertion points in the concrete casting area is 13.

**Figure 10:**
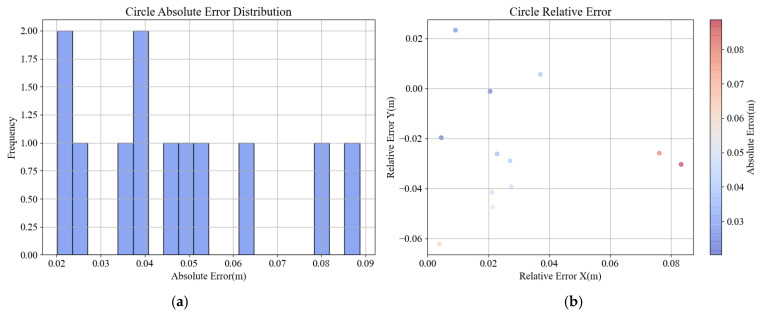

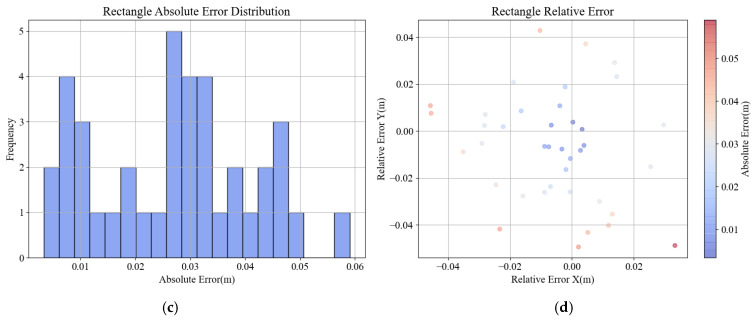
Error histogram and heat map: (**a**) concrete casting area error histogram; (**b**) concrete casting area error heat map; (**c**) concrete vibration area error histogram; (**d**) concrete vibration area error heat map.

The discrepancy between the measured and the true values is depicted in Figure 10, using the maximum and minimum errors and RMSE. The RMSE is the square root of the MSE and is commonly used as a metric to measure the difference between model predictions and actual values. The results are presented in Table 2. As can be seen in Figure 10a,b, the errors in the circular distribution area are primarily between 0.02 m and 0.06 m, with only two points exhibiting larger deviations ranging from 0.08 m to 0.09 m. From Figure 10c,d, it can be observed that the errors in the rectangular vibrating area concentrate between 0 m and 0.05 m, with only one point showing a significant deviation of 0.089 m. As indicated in the Table 2, the minimum error distance in the circular distribution area is 0.02 m, the maximum is 0.089 m, and the overall RMSE is 0.0493 m. The error distance in the rectangular vibrating area ranges from a minimum of 0.003 m to a maximum of 0.059 m, with an overall RMSE of 0.031 m. Therefore, the results indicate that the error between the calculated and the actual value is within 10 cm, and the majority of the errors are below 5 cm. The insertion falls within the error margin, meeting the detection accuracy requirements.

**Figure 11:**
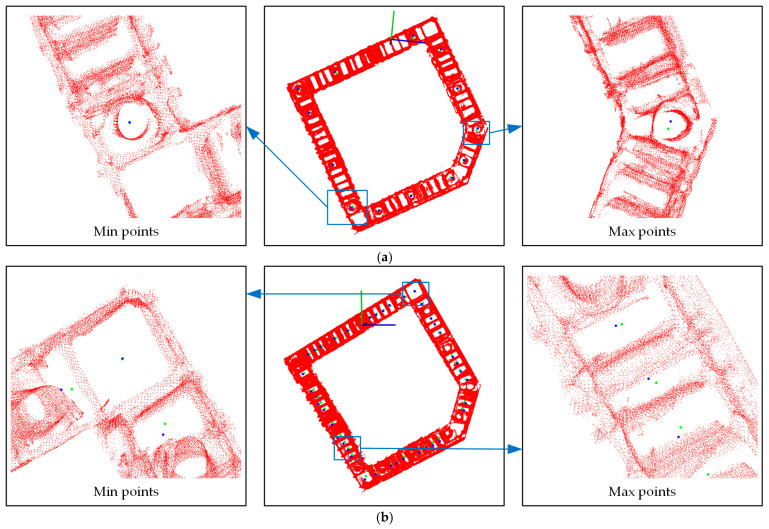
Actual graph of error: (**a**) circle detection results; (**b**) rectangle detection results. (The red axis is the X-axis, the green axis is the Y-axis, and the blue axis is the z-axis).

To facilitate the observation of the discrepancy, the points with true values in the CAD drawing are transformed into a model coordinate system and displayed in Figure 11. The true value points are represented in blue, and the measured points are in green. Among the circular concrete casting points, the group of points with the smallest error nearly overlaps, while the distance between the group of points with the largest error is 0.089 m. This is attributed to the smooth and complete scanning for the group of points with the smallest error, resulting in more accurate calculation results. Conversely, the group of points with the largest error has a larger scanning angle, leading to an incomplete scanned and subsequent deviation of the fitted circle center from the actual concrete casting point.

Similarly, among the rectangular vibration points, the group of points with the smallest error nearly overlaps, whereas the distance between the group of points with the largest error is 0.059 m. The former is also attributed to high-quality scanning, while the latter is affected by scanning angles and obstacles that cause the center of the fitted rectangle to deviate from the actual vibration point.

## 4. Conclusions

The autonomous vibration method for a vibrating robot arm is proposed based on LiDAR 3D feature extraction. The algorithm is less affected by illumination, and the 3D model is restored based on the point cloud. The method employs NDT for coarse matching and ICP for fine matching to stitch together local point clouds. The spliced point cloud data is optimized using the g2o graph optimization method to recreate the overall point cloud model of the actual construction site. Subsequently, by employing the method of 3D projection to a 2D plane and image processing technology, the concrete vibration points and concrete casting points are successfully identified. The MSE obtained by splicing using the proposed algorithm are reduced by 11.5% and 11.37% in comparison to the ICP algorithm and NDT algorithm, respectively. For the rectangular vibration points and circular concrete casting points calculated, the maximum errors are 0.059 m and 0.089 m, respectively, and the RMSEs are 0.031 m and 0.0493 m, respectively. The calculation results fall within the error margin and satisfy the actual insertion accuracy requirements.

This method not only restores the building scene based on NDT, ICP, and g2o but also detects it by dimensionality reduction and image processing methods. The method effectively improves the detection of concrete vibration and pouring position information in construction scenarios. In the future, we need to focus on further solving for a larger range of complex buildings and improving detection accuracy.

## Figures and Tables

**Table 1:** MSEs of different algorithms.

Algorithm	Proposed Algorithm	NDT-ICP	ICP	NDT
MSE	**0.0123949**	0.0139337	0.0140056	0.0139856

**Table 2:** RMSE and maximum and minimum points.

Category	Point	Measure X	Measure Y	True X	True Y	Distance (m)	RMSE (m)
fabric area	Min	−5.7132	−0.7539	−5.7087	−0.7736	0.0202	0.0493
	Max	−2.4523	3.5023	−2.369	3.4719	0.0887	
Vibration area	Min	1.0403	1.4328	1.0435	1.4335	0.0034	0.0310
	Max	−2.9548	−2.3259	−2.9216	−2.3747	0.059	

## Data Availability

Data are contained within the article.
